# A small-molecule pan-class I glucose transporter inhibitor reduces cancer cell proliferation in vitro and tumor growth in vivo by targeting glucose-based metabolism

**DOI:** 10.1186/s40170-021-00248-7

**Published:** 2021-03-26

**Authors:** Pratik Shriwas, Dennis Roberts, Yunsheng Li, Liyi Wang, Yanrong Qian, Stephen Bergmeier, Jennifer Hines, Subhodip Adhicary, Corinne Nielsen, Xiaozhuo Chen

**Affiliations:** 1 Department of Biological Sciences, Ohio University, Athens, OH 45701 USA; 2 Edison Biotechnology Institute, Ohio University, Athens, OH 45701 USA; 3 Molecular and Cellular Biology Program, Ohio University, Athens, OH 45701 USA; 4 Department of Biomedical Sciences, Ohio University, Athens, OH 45701 USA; 5 Department of Chemistry and Biochemistry, Ohio University, Athens, OH 45701 USA; 6 Translational Biomedical Sciences Program, Ohio University, Athens, OH 45701 USA; 7 Edison Biotechnology Institute, Ohio University, 172 Water Tower Drive, Athens, OH 43701 USA

**Keywords:** The Warburg effect, Glycolysis, TCA cycle, Metabolomics, Anticancer therapeutics, docking

## Abstract

**Background:**

Cancer cells drastically increase the uptake of glucose and glucose metabolism by overexpressing class I glucose transporters (GLUT1-4) to meet their energy and biomass synthesis needs and are very sensitive and vulnerable to glucose deprivation. Although targeting glucose uptake via GLUTs has been an attractive anticancer strategy, the relative anticancer efficacy of multi-GLUT targeting or single GLUT targeting is unclear. Here, we report DRB18, a synthetic small molecule, is a potent anticancer compound whose pan-class I GLUT inhibition is superior to single GLUT targeting.

**Methods:**

Glucose uptake and MTT/resazurin assays were used to measure DRB18’s inhibitory activities of glucose transport and cell viability/proliferation in human lung cancer and other cancer cell lines. Four HEK293 cell lines expressing GLUT1-4 individually were used to determine the IC_50_ values of DRB18’s inhibitory activity of glucose transport. Docking studies were performed to investigate the potential direct interaction of DRB18 with GLUT1-4. Metabolomics analysis was performed to identify metabolite changes in A549 lung cancer cells treated with DRB18. DRB18 was used to treat A549 tumor-bearing nude mice. The *GLUT1* gene was knocked out to determine how the KO of the gene affected tumor growth.

**Results:**

DRB18 reduced glucose uptake mediated via each of GLUT1-4 with different IC_50_s, which match with the docking glidescores with a correlation coefficient of 0.858. Metabolomics analysis revealed that DRB18 altered energy-related metabolism in A549 cells by changing the abundance of metabolites in glucose-related pathways in vitro and in vivo. DRB18 eventually led to G1/S phase arrest and increased oxidative stress and necrotic cell death. IP injection of DRB18 in A549 tumor-bearing nude mice at 10 mg/kg body weight thrice a week led to a significant reduction in the tumor volume compared with mock-treated tumors. In contrast, the knockout of the *GLUT1* gene did not reduce tumor volume.

**Conclusions:**

DRB18 is a potent pan-class I GLUT inhibitor in vitro and in vivo in cancer cells. Mechanistically, it is likely to bind the outward open conformation of GLUT1-4, reducing tumor growth through inhibiting GLUT1-4-mediated glucose transport and metabolisms. Pan-class I GLUT inhibition is a better strategy than single GLUT targeting for inhibiting tumor growth.

**Supplementary Information:**

The online version contains supplementary material available at 10.1186/s40170-021-00248-7.

## Background

Cancer is a leading cause of disease-related deaths in the USA. It is estimated that in 2020 alone 1,735,350 new cases of cancer will be diagnosed in the USA, and 609,640 people are expected to die from the disease (https://www.cancer.gov/about-cancer/understanding/statistics). Cancer is not only a group of genetic-related diseases but also metabolic diseases. Otto Warburg, in the 1930s, made a series of observations on how cancer cells utilize glucose for altering their metabolism to sustain, survive and grow [1]. Glucose-based cancer metabolism research has been revived in the last two to three decades partly due to a phenomenal increase in cutting-edge molecular biology techniques and realization of the important roles played by the Warburg effect in cancer biology and cancer metabolism [2, 3]. Because cancer cells divide and multiply rapidly, they are in constant need of nutrients. Glucose is one of these nutrients whose uptake is drastically upregulated in cancers [4]. Cancer cells are known to heavily depend on glucose and are addicted to it [5]. Glucose is utilized by cancer cells, not only for energy generation but also for biomass synthesis and maintenance of redox balance [6, 7]. Cancer cells are known to be more sensitive and vulnerable to changes in glucose supply than normal cells [8]. It is well-documented that glucose deprivation results in cancer cell death; thus, targeting glucose uptake in cancer cells becomes an attractive anticancer strategy [9].

Glucose is primarily taken up by cancer cells via membrane-spanning proteins called glucose transporters (GLUTs). GLUTs belong to a homologous family of 14 uniporter transporter proteins. Among these GLUT1-4, or class I GLUTs, have been extensively studied, and have been found to possess conserved structures and to be most upregulated in and be relevant to cancers [10]. GLUT1 is expressed in a majority of cancer types such as lung, breast, and melanomas, among others [11], while GLUT3 is found to be upregulated in glioblastomas [12]. GLUT2 is found in liver and pancreatic cancers and GLUT4 in multiple myelomas as well as head and neck cancers [13, 14]. However, in general, GLUT1-4 are expressed at different levels and in different ratios to each other in most cancer cells of different cancer types. GLUTs were explored as an anticancer therapeutic target previously. GLUT inhibitors have been isolated from natural products, as well as synthetically derived [14, 15]. For single GLUT or multi-GLUT targeting, GLUT inhibitors could be divided into two types: GLUT-specific or pan-GLUT inhibitors. The majority of GLUT inhibitors have been studied as single GLUT targeting molecules although later studies have shown that they may also target other GLUTs. BAY876, STF-31, Fasentin, and oximes among others have shown anticancer efficacy against many cancer types while selectively targeting GLUT1 in vitro and/or in vivo [16–19]. Phloretin is a GLUT2 inhibitor while Glutor is a GLUT1/3 inhibitor [20, 21]. Ritonavir and compound 20 are inhibitors of GLUT4 [22]. However, cancer cells are known to express more than one type of GLUT. For example, ZR-75 breast cancer cells express GLUT1-4 [23]. Furthermore, some cancers use different GLUTs for different functions. Lung cancer cells, for instance, use GLUT1 for proliferation and growth while GLUT4 is used for metastasis, and GLUT3 is upregulated and is responsible for liver metastasis from primary colorectal cancer [24, 25].

As a result, it sometimes becomes necessary or even advantageous to develop pan-class I GLUT inhibitors which simultaneously target multiple GLUTs to further enhance their glucose transport inhibitory activity and anticancer efficacy. So far, at least in part due to deficiencies in required technology, the development of pan-class I GLUT inhibitors has been rare and no in vivo comparison between pan-class I GLUT and single GLUT inhibitors in their relative tumor growth reduction efficiency has been made.

Previously, we designed synthetically derived inhibitors of GLUTs based on natural products from tannins [25, 26]. WZB117, the first-generation lead compound, was characterized both in vitro and in vivo as an anticancer agent [26] and subsequently reviewed as a representative GLUT1-inhibitor [27, 28]. Our lab and others have been tested WZB117 successively in many cancer types and have shown successful GLUT targeting and anticancer efficacy [29–33]. However, WZB117 is not stable under physiological conditions due to the presence of ester bonds, limiting its ability to be developed into an anticancer therapeutic. We hypothesized that pan-GLUT inhibitors with higher physiological stability would significantly improve their anticancer efficacy in cancer types that express multiple GLUTs. In this study, we report mechanistic studies on a novel inhibitor DRB18, which we recently synthesized [34]. Cell viability assays were used to determine its cancer cell growth-inhibitory activities. Four single GLUT-expressing cell lines, coupled with docking studies, were used to determine the selectivity of the compound in GLUTs for the glucose uptake inhibitory activity and potential interactions between the compound and GLUT1-4. We used cancer cell lines, nude mice with A549 tumors or GLUT1-deficient A549 tumors, and metabolomics analysis was used to identify our compound’s anticancer efficacy and mechanisms in vitro and in vivo as a pan-class I GLUT inhibitor. The results of this study may have profound and broad implications in the future development of GLUT inhibitors as more effective anticancer therapeutics.

## Methods

### Compound inhibitors and other chemicals

Compound WZB117 and DRB18 were synthesized as previously reported [25, 34]. Compound solutions were freshly prepared by dissolving the compounds in dimethyl sulfoxide (DMSO) before each experiment. Chemicals 5-(N-ethyl-N-isopropyl) amiloride **(**EIPA, a macropinocytosis inhibitor) and chlorpromazine hydrochloride (a clathrin-mediated endocytosis inhibitor) were purchased from Sigma-Aldrich.

### Cell lines, cell culture, and experimental controls

Human non-small cell lung cancer (NSCLC) cell lines A549 and H1299, human cervical cancer Hela were purchased from American Type Culture Collection (ATCC). A549 and H1299 cells were cultured in standard Dulbecco’s Modified Eagle Medium (DMEM) with 10% fetal bovine serum and 1% penicillin/streptomycin in an incubator with 5% CO_2_ at 37 °C. Hela cells were cultured in similar conditions except that DMEM was replaced by Eagle’s Minimum Essential Medium [34]. Cells were treated with compound DRB18 for 48 or 72 h. DRB18 (5 or 10 μmol/L) was used in the experiments unless otherwise specified. Mock (DMSO)-treated samples served as negative controls.

### Glucose uptake assay in cancer cells and in specific GLUT-expressing cell lines

The inhibitory activity of DRB18 on glucose transport in cancer cells was analyzed by measuring the cell uptake of 2-deoxy-d-[^3^H] glucose as previously described [26, 34].

For four GLUT-specific cell lines, HEK293 cells expressing one specific GLUT only per cell line [35] were a gift from Dr. Paul Hruz at Washington University of St. Louis. Cell plates were pre-treated with 25 μg/ml Polyethyleneimine for 20 min. The plates were then allowed to dry for 5–10 min after aspiration of PEI solution. A total of 50,000 cells were seeded into each well of the 24-well plate. After overnight incubation, cells were washed with KRP buffer twice and 225 μl of glucose-free KRP buffer was added. Additionally, mock or inhibitor was added to cells and incubated for 30 min. A mixture composed of 12.5 μl of 37 MBq/l 2-deoxy-D-[^3^H] glucose and 25 μl of 1 mmol/l regular glucose was added to cells to initiate glucose uptake. After 4 min, the cells were washed and lysed, and then radioactivity of the cell lysates was counted using a LS 6500 Scintillation Counter (Beckman Coulter). Glucose uptake assays in cancer cell lines were performed as previously described [25, 34].

### Cell proliferation assays

Cell proliferation assays were performed using resazurin as a colorimetric dye. Briefly, 2,500 cells were seeded into a 96-well plate. The cells were treated with appropriate compound concentrations or mock for 48 or 72 h. Resazurin was then added for 20 min. Absorbance values were measured at E_x_ 560 nM and E_m_ 590 nM. Values of mock-treated samples were used as controls to normalize all the data points.

### Protein docking studies

A 2D molecular model of DRB18 was constructed using an NIH PubChem Sketcher (https://pubchem.ncbi.nlm.nih.gov/edit3/index.html). The 3D model of the compound was constructed by importing the 2D model into the 2D sketcher (Schrödinger). A 4PYP model for hGLUT1 inward open conformation and 5C65 model for hGLUT3 outward open conformation were used to generate homology models for other GLUTs in the respective conformations using SWISS-MODEL [35, 36]. A protein preparation wizard module (Schrödinger) was used to prepare the GLUTs for docking, and receptor grid preparations were conducted using the Glide module of Maestro (Schrödinger) with default protocols [31, 37, 38]. Grid box settings including amino acids for the centroid of the grid box have been discussed in detail in supplementary methods. DRB18 was initially docked using Standard Precision mode in Glide. The pose with the lowest Glide score was then used to redock DRB18 using the Induced-fit docking module in Glide. The resultant best docked structure for the compound was selected based on the Glidescore values.

### Seahorse studies

An XFe 24 Extracellular Flux Analyzer (Seahorse Bioscience) was used for performing metabolic analyses. Briefly, glycolysis-related extracellular acidification rate (ECAR) or mitochondrial OXPHOS related oxygen consumption rate (OCR) of 30,000 A549 cells treated with DMSO or with DRB18 (10 μM), macropinocytosis inhibitor EIPA (40 μM), or chlorpromazine hydrochloride (25 μM) were measured continuously with the analyzer. First, the cells were seeded in DMEM media. After overnight incubation, the media was removed and cells were washed with KRP buffer. The cells were treated with 10 mM glucose in KRP for 1 h. Following this, the cells were incubated in the analyzer for 30 min and compounds or compound mixtures were added. A549 cells treated with DMSO served as no-treatment control.

### ATP measurement study

Intracellular ATP concentration was measured using an ATPlite luminescence ATP detection assay system from Perkin-Elmer as previously described [26, 29]. Briefly, cells were seeded at a density of 4,000 cells in each well of a 96-well plate. ATP levels were measured after 72 h of treatment with different concentrations of DRB18 or mock (DMSO). Protein concentration of cells in each well was determined for both lactate and ATP measurements for signal normalization [34].

### Cell cycle analysis and apoptotic and necrotic assay

Cell cycle was analyzed as previously described [26, 34]. For identification of apoptotic and necrotic cells, the treated cells were stained with 7-AAD and Apoxin-V according to the manufacturer’s instructions (Abcam; Apoptosis/Necrosis Assay Kit) and then subjected to flow cytometric analysis.

### Intracellular ROS measurement

A549 cells were seeded in a 96-well plate. Cells were treated with DRB18 for 72 h and then washed with PBS twice. Then, the cells were treated with DCFDA dye for 30 min before reading according to the company-provided protocol (Abcam; DCFDA/H2DCFDA-Cellular ROS Assay Kit).

### Western blot analysis

Western blot analyses were conducted using the standard protocol [39]. Antibodies for GLUT1-4 were used as described above. Cofilin (Cell Signaling Technology-5175) was used as a loading control.

### Animal study

All animal studies described below were conducted in accordance with US government regulation on animal care and Ohio University IACUC-approved protocols.

Male *NU/J* nude mice of 3 to 4 weeks of age were purchased from The Jackson Laboratory and were fed with the Irradiated Teklad Global 19% protein rodent diet from Harlan Laboratories. The protocol for cell injection, treatment administration, weekly tumor measurement, animal euthanasia, and final tumor measurements were performed as described previously (unless stated otherwise) [31]. Tumor cell-injected mice were randomly divided into 2 groups: control group (*n* = 10) treated with PBS/DMSO (1:1, v/v) and 10 mg/kg (body weight) DRB18 treatment group (*n* = 10) dissolved in PBS/DMSO solution (1:1, v/v). Mice were given an intraperitoneal injection with either PBS/DMSO vehicle or compound DRB18 (10 mg/kg) thrice a week for 5 weeks.

### *GLUT1* gene knockout in A549 cells and KO tumor study

*GLUT1* gene knockout was performed using as described previously [40]. The gRNA containing 20-nucleotide target sequences of GLUT1 (3′-CTTCGTGTCCGCCGTGCTCA-5′) was purchased from GenScript (Piscataway, NJ).

For the in vivo tumor growth studies for Wildtype and GLUT1KO A549 cells, the protocol followed was as described previously (unless otherwise stated) [31, 40]. Male nude mice of *NU/J* strain of 5 weeks of age were purchased from the Jackson Laboratory (Bar Harbor, ME) and were maintained under specific pathogen-free conditions. Wildtype or *GLUT1* knockout (KO) A549 injected mice were euthanized 4 weeks after tumor cell injections and tumors were surgically removed, weighed, and photographed.

### LC-MS/MS metabolomics-metabolite extraction and sample preparation

5 × 10^6^ A549 cells were treated with or without 10 μM DRB18 (*n =* 3) for 48 h and then prepared as described previously ([41], supplementary methods). For tumor samples, 100 mg of tumors were obtained from vehicle- and DRB18-treated mice (*n =* 4) *s*amples were used from each group. Tumors were homogenized in a Beadbud homogenizer (Benchmark scientific) in a mixture of LC-MS grade ice-cold methanol and water (1:1; v/v). The supernatant was collected and sonicated in a water bath incubator for 15 min, followed by centrifugation at 13,000 rpm for 10 min and collection of the supernatant. Supernatants collected from in vitro and in vivo extraction were then lyophilized using a speed vacuum evaporator. The samples were then dissolved into a mixture of LC-MS grade acetonitrile/water (1:1; v/v) for analysis.

### LC-MS/MS metabolomics-LC-MS/MS experiment and analysis

The entire LC-MS/MS experiment was performed in the Campus Chemical Instrumentation Center at The Ohio State University. An untargeted metabolomics approach was used by utilizing Agilent Q-TOF 6545 mass spectrometer connected to an Agilent 1290 UHPLC system with a Poroshell 120 SB-C18 (2 × 100 mm, 2.7-μm particle size) column. Data acquisition (Masshunter, Agilent Technology) and peaks integration (Progenesis, Agilent Technology) was followed by compound identification using both XCMS as well as Metaboanalyst 4.0 software. Peak areas were normalized using internal standards and were subjected to relative quantification analyses with control (DMSO) for in vitro analysis and control (vehicle-treated tumors) for in vivo analysis.

### Data analysis of metabolomics data

Data acquisition was performed using Masshunter software (Agilent Technology), and peaks were integrated by using Progenesis software (Agilent Technology). Compound identification was performed using both XCMS as well as Metaboanalyst 4.0 software. Peak areas were normalized using internal standards and were subjected to relative quantification analyses with control (DMSO) for in vitro analysis and control (vehicle-treated tumors) for in vivo analysis.

### Immunofluorescence study of tumor sections

Fresh frozen xenograft tumors were cryopreserved in 30% sucrose solution before being embedded in OCT (Tissue-Tek). 14 μm thick tumor sections (Leica CM1950 Cryostat) were fixed in 2% PFA, blocked, and permeabilized in 5% donkey serum (Jackson ImmunoResearch)-0.1% Triton-X-1X PBS solution, before incubation with primary antibodies, overnight at 4 °C. Primary antibodies used were: rabbit anti-GLUT1 (1:400, Novus-NB110-39113), rabbit anti-GLUT2 (1:400, Novus-NBP2-22218), rabbit anti-GLUT3 (1:400, Abclonal-A8150), rabbit anti-GLUT4 (1:400, BIOSS-bs038-FR), mouse anti-Ki67 (1:300, Novus-NBP2-22112). Signal detection was achieved using goat anti-mouse-Alexa Flour-647 (1:1000, Cell Signaling) and goat anti-rabbit-Alexa Fluor-488 (1:1000, Cell signaling). Sections were mounted with ProLong Gold Antifade Mountant with DAPI (ThermoFisher Scientific). Images were acquired on a Nikon Eclipse Ni-U epifluorescent microscope with consistent intensity and exposure parameters and analyzed using NIS-Elements program. Experiments were conducted on *n* = 4 tumors each for vehicle and DRB18 with appropriate negative controls.

### Statistical analysis

For all the cell and molecular studies, each experimental condition was performed in triplicates, quadrapulates, or hexads (or as mentioned otherwise), and the experiment was repeated at least once. Data is reported as mean ± standard deviation and analyzed using Student’s *t*-test or one-way ANOVA whichever is appropriate. *P <* 0.05 was considered significant.

## Results

We previously showed that WZB117 was a GLUT1-inhibitor capable of inhibiting glucose uptake in GLUT1-only expressing human erythrocytes. It was also able to reduce glucose uptake in different cancer types in vitro and in vivo [30, 31]. Further studies on this compound showed that it acts as a pan-class I GLUT inhibitor targeting GLUT1, 3, and 4 [32]. However, WZB117 is chemically unstable. This study characterizes a novel pan-GLUT inhibitor, DRB18, as a more stable and potent anticancer therapeutic of second generation [34].

### DRB18 is a more stable and more potent pan-GLUT inhibitor against GLUT1-4

**Fig. 1:**
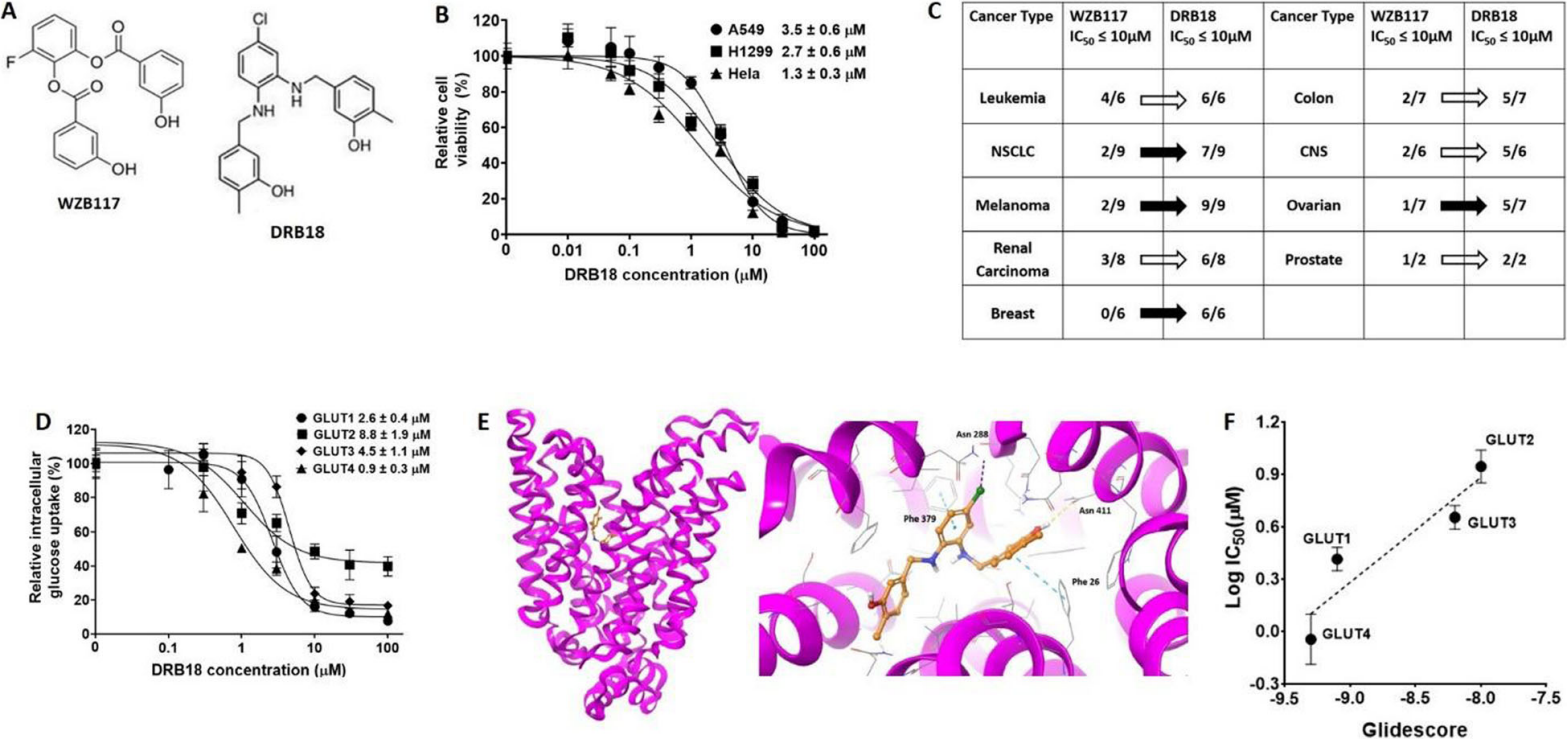
DRB18 is a pan-GLUT inhibitor with multi-GLUT and multi-cancer targeting potential. DRB18 is a lead compound derived from WZB117. It was tested in 9 different cancer types in the NCI60 cell line panel. The protein structure for hGLUT1 was generated using homology modeling from the crystal structure of hGLUT3 PDB-ID 5C65 for the docking study. All values are relative to controls. Filled arrows indicate extremely sensitive cancer types to DRB18 compared with WZB117 and unfilled arrows indicate cancer types with mildly improved potency of DRB18 compared with WZB117. Assay values represented are mean ± SEM. *P ≤ 0.05, **P ≤ 0.01, and ***P ≤ 0.001. **a** Structure of WZB117 and DRB18. **b** DRB18 reduced cell viability in three cancer cell lines after 72 h of treatment in a dose-dependent manner in a resazurin assay. **c** NCI for screening result for anticancer potency of WZB117 and DRB18 in their NCI60 cell line panel. It shows, treated by either WZB117 or DRB18, the number of cancer cell lines in the total number of cancer cell lines in a given cancer type (M/N) that exhibit IC_50_ values lower than 10 μM. The screening was conducted with a cell viability MTT assay at NCI. **d** The IC_50_ determination of DRB18’s glucose uptake inhibitory activity in four different HEK293 cell lines each expressing a single GLUT. **e** DRB18 binds to hGLUT1 binding pocket in outward open conformation. DRB18 forms hydrogen bonds (Asn 411), π-π interactions (Phe 26 and Phe 379), and halogen bonds (Asn 288) with different residues in hGLUT1. The protein is shown with the cytoplasmic side down. Hydrogen bonds and Halogen bonds are shown in broken yellow and purple lines. π-π interactions are shown in broken blue lines. The specific elements are shown in the respective colors with oxygen in red; nitrogen in blue and hydrogen in white. Carbons in DRB18 are shown in yellow and chlorine in dark green. **f** The correlation between the glidescores of DRB18 in hGLUT1-4 docking study (as shown in **e**) and the glucose uptake assay results (IC_50_s in **d**), The correlation coefficient was calculated to be *R*^2^ = 0.8577

DRB18 (Fig. 1a right) is a rationally designed second-generation lead compound-based on the structure of WZB117 (Fig. 1a left). The ester bonds of WZB117 are replaced by stronger/more rigid amine bonds in DRB18, making it much more stable than WZB117. Further studies also found DRB18 reduced cell viability in a dose-dependent manner in three cancer cell lines (Fig. 1b). The compounds were then sent to the National Cancer Institute (NCI) for screening against a panel of 60 different cell lines which belong to nine major cancer types. DRB18 exhibited a much more improved potency against these cancer types compared with WZB117 (Fig. 1c). For example, in the melanoma group, DRB18 exhibited IC_50_ values lower than 10 μM in all nine melanoma cell lines while WZB117 showed such IC_50_s in just two cell lines. Such improved activity was also observed in other cancer types such as NSCLC, breast, and ovarian cancers, among others (Fig. 1c). In some selective cancer cell lines, the IC_50_ values of DRB18 reached a high nM range (unpublished data).

### The docking study results correlate with the IC_50_ study in single GLUT-expressing cell lines

Four HEK293 cell lines that express GLUT1-4 with only one unique GLUT per cell line were used to determine the GLUT selectivity of DRB18. These cells have been used previously for assessing the selectivity of compounds for hexose transporter pfHT in *Plasmodium falciparum* against human GLUTs [35, 42]. DRB18 reduces glucose uptake in these cell lines in a dose-dependent manner with IC_50_s varying from ~ 900 nM to ~ 9 μM (Fig. 1d). This result indicates that DRB18 is a pan-class I GLUT inhibitor selectively reducing glucose uptake via hGLUT1-4.

Next, we hypothesized that this inhibition is due to direct interactions between DRB18 and hGLUTs and a docking study was performed to test the hypothesis. The docking study revealed that DRB18 binds to the outward open conformation of hGLUT1 (Fig. 1e). It interacts with hGLUT1 in multiple ways including hydrogen bonds (Asn 411), π-π interactions (Phe 26 and Phe 379), and halogen bonds (Asn 288) (Fig. 1e). Similarly, DRB18 interacts with amino acid residues located in the central channel region of GLUT2-4 (Supplemental Figure S1). Figure 1f provides a detailed description of the experimental glucose transport inhibitory activities (IC_50_s values) versus virtual binding affinities (glidescores) of DRB18. We performed docking on both the outward open and inward open conformations of GLUTs.

The correlation coefficient for the outward open conformation was *R*^2^ = 0.8577 (Fig. 1f), while the correlation coefficient for the inward open is *R*^2^ = 0.1451 (Supplemental Figure S2). These results led us to conclude that DRB18 is much more likely to bind to the outward open conformation of hGLUTs.

### DRB18 rapidly and potently inhibits glucose transport and glucose metabolism in several cancer cell lines

**Fig. 2:**
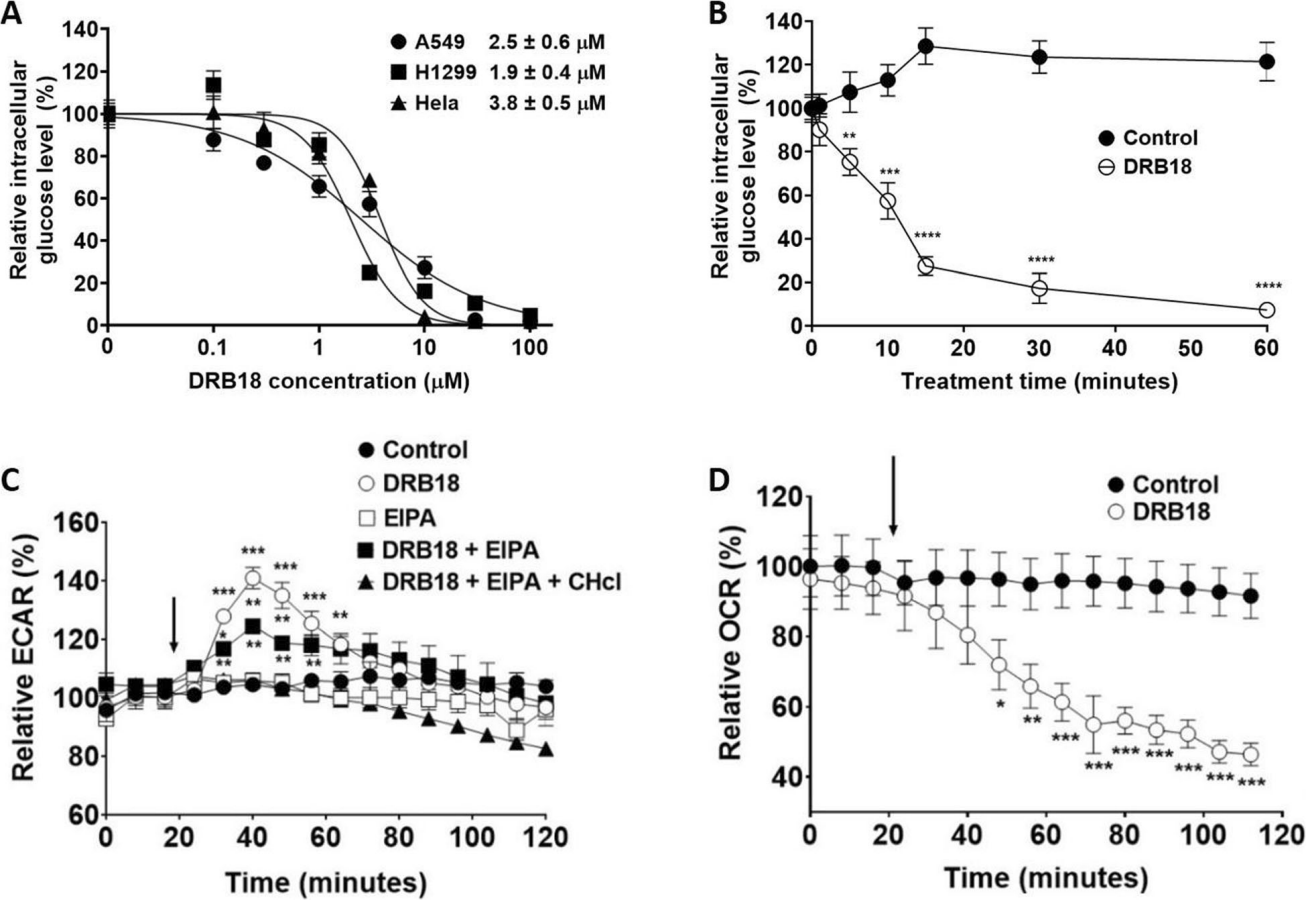
DRB18 rapidly inhibits glucose uptake and glucose metabolism. DRB18’s glucose transport inhibitory activity was investigated by glucose uptake and Seahorse assays. The potential involvement of macropinocytosis in Seahorse analysis was explored. All values are relative to controls. Values represented are mean ± SEM. *P ≤ 0.05, **P ≤ 0.01, and ***P ≤ 0.001. **a** DRB18 reduced [^3^H]-2-deoxy-glucose uptake in a dose-dependent manner in A549, H1299, and Hela cancer cells. **b** DRB18 (10 μM) reduced [^3^H]-2-deoxy-glucose uptake in a rapid and time-dependent manner in A549 cells. **c** DRB18 increased ECAR temporarily in A549 cells. The increase was reduced when a macropinocytosis inhibitor EIPA was added at the time pointed by the arrow. The increase in ECAR was completely neutralized when clathrin-mediated endocytosis inhibitor Chlorpromazine hydrochloride was used along with DRB18 and EIPA. **d** OCR is reduced when A549 cells are treated with DRB18 in comparison with control (DMSO)

Next, we examined DRB18’s acute activity in vitro in cancer cells. We hypothesized that, if DRB18 reduced glucose uptake in GLUT-specific HEK-293 cell lines, then it should also do the same in cancer cells, as these cancer cells express GLUT1-4 (https://www.proteinatlas.org/). Glucose uptake assay showed that DRB18 reduced glucose uptake in A549 and H1299 (NSCLC) and Hela (cervical cancer) cells in a dose-dependent manner with IC_50_ values ranging from 1.9 to 3.6 μM (Fig. 2a). In a second time-dependency glucose uptake assay, DRB18 reduces glucose uptake after 1 min of treatment compared with that of time zero, suggesting that inhibition is rather rapid and possibly direct (Fig. 2b). To investigate how glucose uptake inhibition by DRB18 would affect basal ATP production, a Seahorse analysis was performed in the presence and absence of DRB18. During this experiment, glucose was used as the only source of energy. It was expected that DRB18 treatment would reduce ECAR. However, DRB18 led to a relatively large but temporary increase in ECAR under these conditions (Fig. 2c). We investigated whether this could be due to endocytosis as an alternative mechanism of glucose uptake. When DRB18 was added with EIPA, a macropinocytosis inhibitor [43], the increase in ECAR was reduced compared with DRB18 alone (Fig. 2c). Further addition of chlorpromazine hydrochloride (CHcl), an inhibitor of clathrin-mediated endocytosis, along with DRB18 and EIPA, completely eliminated the temporary ECAR increase. These results suggest that glucose could be taken into A549 cancer cells via GLUTs or macropinocytosis or clathrin-mediated endocytosis [44]. To validate this finding, we repeated the glucose uptake assay in the same conditions as ECAR and obtained similar results (supplemental Figure S3). OCR was reduced in DRB18 treated A549 cells (Fig. 2d) suggesting that DRB18 treatment led to a decrease in both glycolysis and TCA cycle.

### Metabolomics analysis revealed that DRB18 inhibits multiple metabolic pathways associated with glucose metabolism

**Fig. 3:**
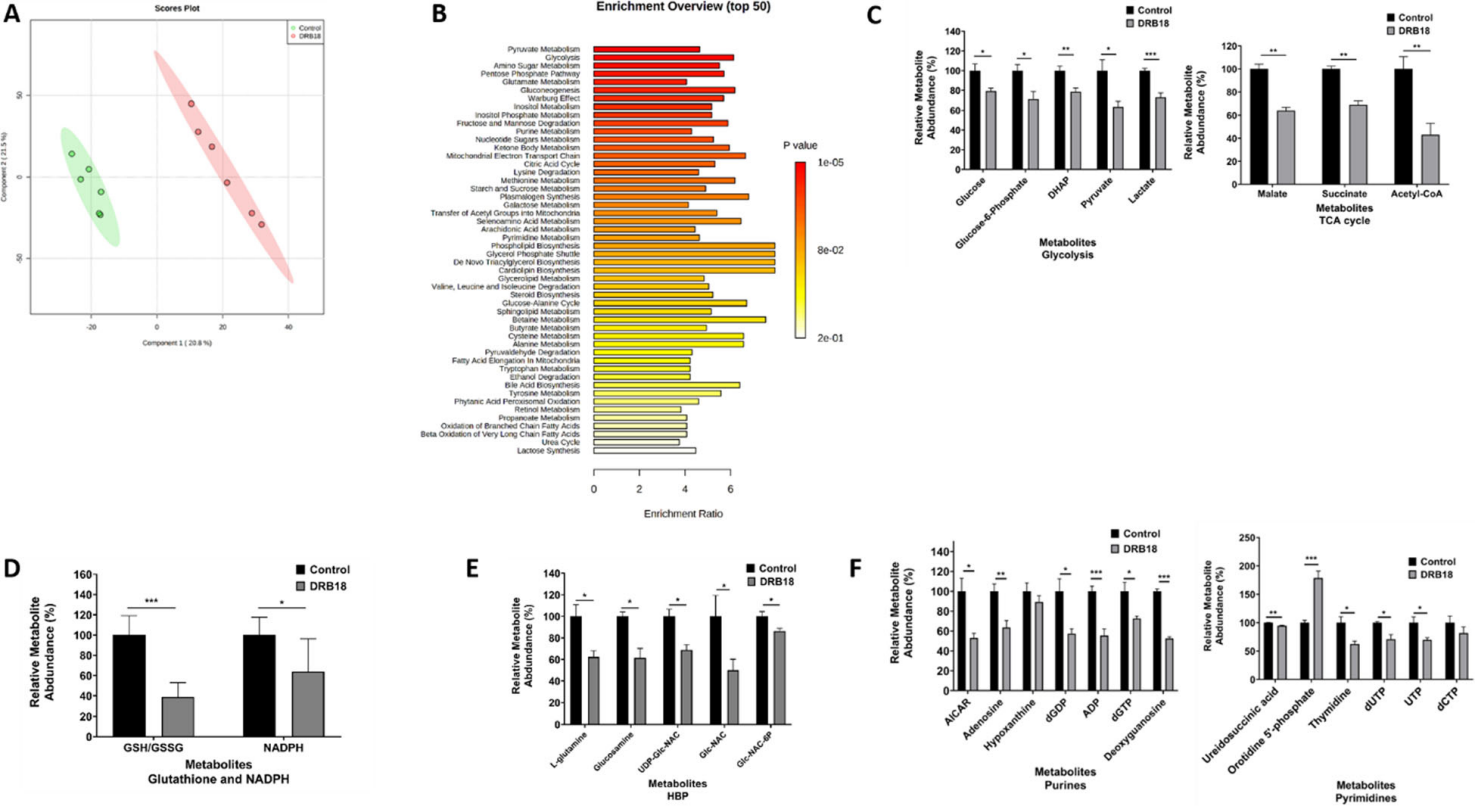
Metabolomics study to determine effects of DRB18 in A549 cells. A549 cells were treated with control (DMSO) or 10 μM DRB18 for 48 h and polar metabolites were extracted and analyzed by performing LC-MS/MS mass spectrometry. Peak areas integrated using Progenesis QI (Agilent Corporation) were normalized. XCMS was used for metabolite identification. MSEA and PLS-DA analysis was performed using utilizing Metaboanalyst 4.0. Statistical analysis was performed using Graphpad 8 software. All values are relative to controls. Values represented are mean ± SEM. *P ≤ 0.05, **P ≤ 0.01, and ***P ≤ 0.001. **a** PLS-DA analysis of metabolites in comparison between control and DRB18. **b** Metabolite set enrichment analysis for carbon-based metabolites in A549 cells treated with or without DRB18. **c** DRB18 reduced metabolites in glycolysis and TCA cycle, which are the primary source of ATP production. **d** DRB18 altered GSH/GSSG ratio and reduced NADPH levels which are related to increased redox imbalance and oxidative stress. **e** DRB18 treatment altered purine and pyrimidine metabolism. **f** DRB18 altered abundances of metabolites responsible in hexosamine biosynthetic pathway (HBP) which related to the reduction in protein glycosylation

Glucose is known to contribute to central metabolism/the Warburg effect in cancer cells and glucose deprivation is toxic to tumors [43, 45]. Thus, we hypothesized that glucose-dependent metabolism in cancer cells would be affected by DRB18 and performed a comprehensive untargeted metabolomics analysis in A549 cells treated with or without DRB18. DRB18 (10 μM) was used for treating cancer cells for 48 h. The duration was kept at 48 h to determine how altered DRB18-mediated metabolism led to cell death after 72 h. PLS-DA principal component analysis revealed that metabolites in DRB18- and control- (DMSO) treated A549 cells were separated into components 1 and 2 (Fig. 3a). The enrichment analysis revealed the key metabolic pathways affected by DRB18 treatment include amino sugar metabolism, the key pathway for glycosylation of GLUTs, as well as many glucose-dependent pathways such as the TCA cycle, and pyrimidine metabolism, among others (Fig. 3b).

Glycolysis and the TCA cycle are the metabolic processes that directly depend on glucose uptake in cancer cells, so we tested if DRB18 altered the metabolites in these pathways [46]. As shown in Fig. 3c, DRB18 reduced the abundance of metabolites in both glycolysis and the TCA cycle, suggesting that glucose uptake inhibition in A549 cells leads to a decrease in the productivity rates of these processes. NADPH is a key metabolite responsible for maintaining redox homeostasis in cancer cells. It is used to maintain reductive capacity in tumors and its reduction results in oxidative stress. GSH and GSSG are responsible for maintaining redox homeostasis in cancer cells. The alteration in their ratio is a marker for oxidative stress. DRB18 treatment led to a decrease in NADPH levels, as well as a decrease in the GSH/GSSG ratio, suggesting that DRB18 treatment resulted in an increase in oxidative stress in A549 cells (Fig. 3d). Figure 3e shows that DRB18 changed the abundance levels of metabolites in the purine-pyrimidine synthesis pathway suggesting that DNA synthesis is also reduced. GLUTs are active when they are glycosylated. It was found that DRB18 reduced expression of glycosylation-required metabolites and thus reduced the glycosylating capacity of A549 cells to keep GLUTs active (Fig. 3f).

**Fig. 4:**
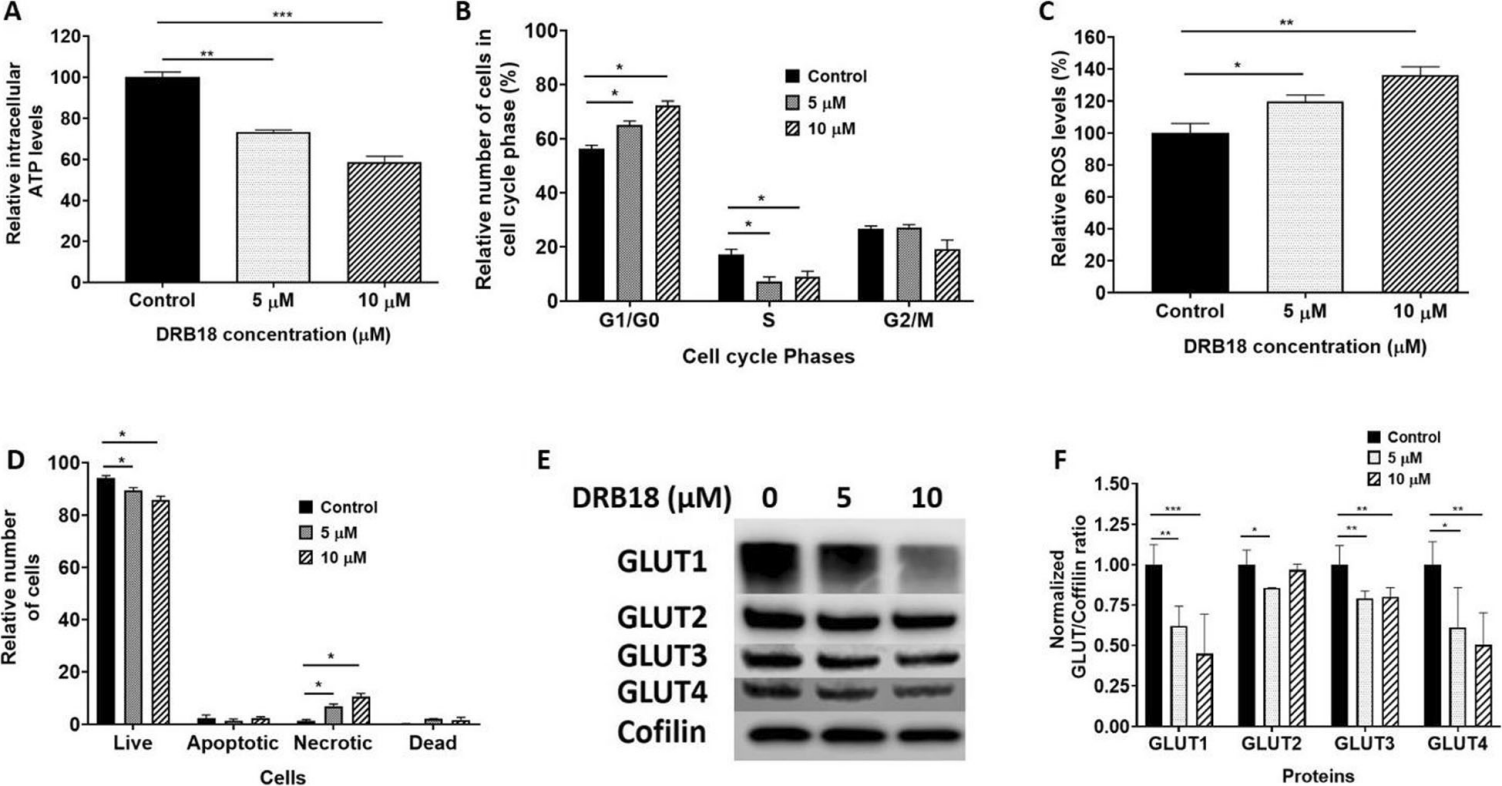
Chronic effects of DRB18 in A549 cancer cells (72 h). The chronic effects of the DRB18 treatment were investigated by using ATP assay, cell cycle analysis, ROS assay and western blot in 72-h-treated cells. All values are relative to mock-treated controls. Values represented are mean ± SEM. *P ≤ 0.05, **P ≤ 0.01, and ***P ≤ 0.001. **a** DRB18 reduced intracellular ATP levels in A549 cells. **b** DRB18 increased the percentage of cells in the G1 phase of the cell cycle and reduces them in the S phase. **c** DRB18 induced the increase in ROS levels. **d** DRB18 induced the increase in the number of necrotic cells in flow cytometry analysis. **e** DRB18 treatment reduced expression of GLUT1-4 at the protein level. **f** Quantification of western blot results is shown in (**e**). Cofilin was used a control

DRB18 treatment for 72 h led to a dose-dependent decrease in ATP levels (Fig. 4a). This is consistent with the Seahorse and metabolomics study results, suggesting that the reduction in ATP production is via glucose transport and glucose metabolism pathways.

### DRB18 treatment led to G1/S phase arrest and increased oxidative stress in A549 cells

In the cell cycle study, DRB18 treatment led to an increase in cells in the G1 phase compared with those in the S phase suggesting that DRB18 treatment causes cell cycle arrest in the G1/S phase transition (Fig. 4b; supplemental figure S4). DRB18 increased ROS levels in A549 cells suggesting that induced oxidative stress is likely due to the decrease in GSH/GSSG ratio as shown by metabolomics data (Fig. 4c). DRB18 was found to cause cell death via necrosis (Fig. 4d; supplement Figure S4), consistent with WZB117 [31]. Nutrient deprivation (glucose starvation), oxidative stress (increase in ROS), and energy depletion (reduction in ATP), working together, ultimately led to cell death in the form of necrosis [47]. Western blot analysis revealed that indeed DRB18 treatment reduced expression of glycosylated GLUT1 (appearing as a smear band, since GLUT is a glycosylated protein) and GLUT2-4 in A549 cells in a dose-dependent manner (Fig. 4e and f). This data indicates that DRB18 reduces the abundance of mature and active GLUT1-4 in A549 cells.

### DRB18 treatment resulted in a significant reduction in tumor volume in tumor-bearing nude mice

**Fig. 5:**
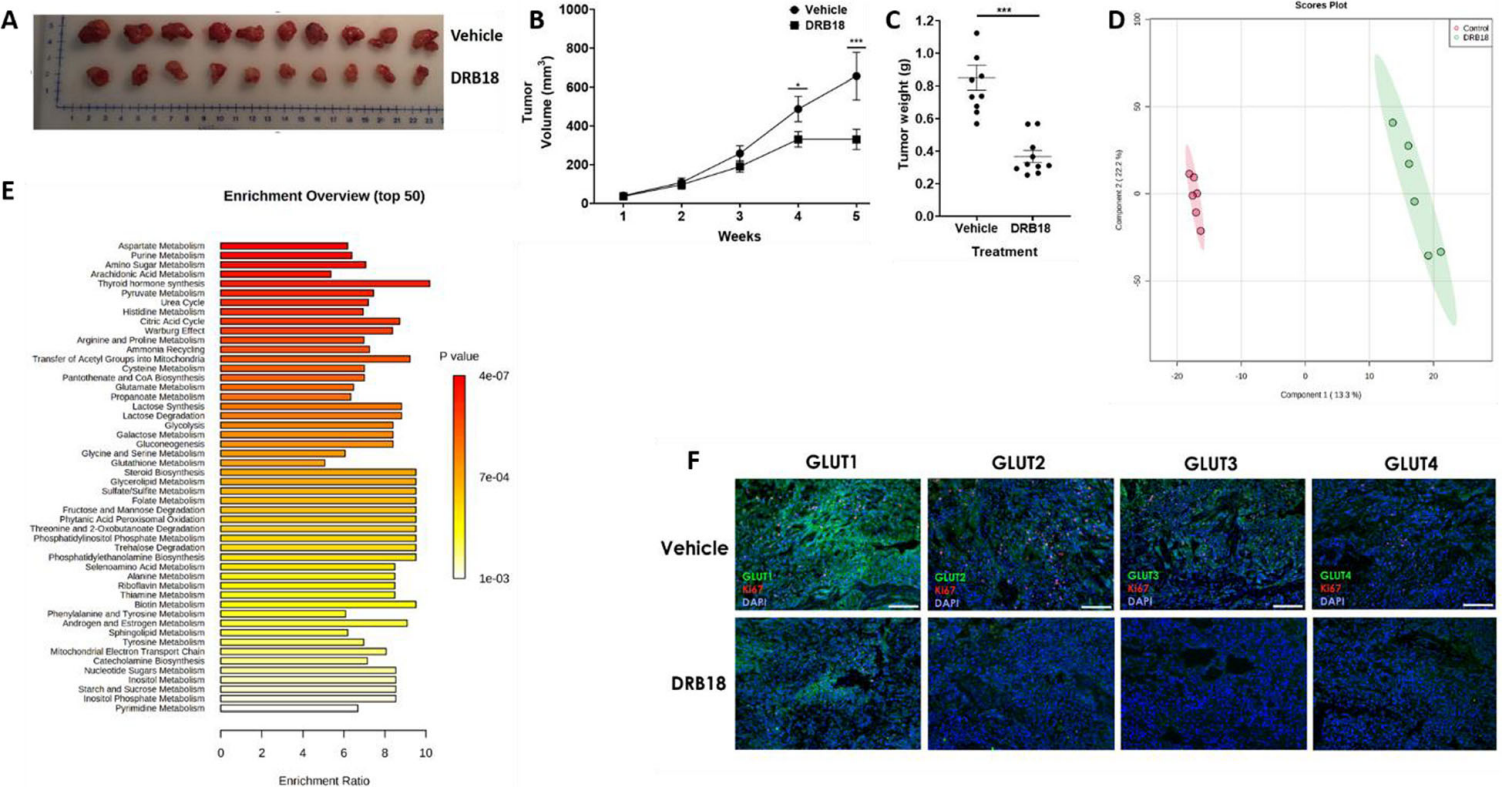
DRB18 inhibited the growth of A549 tumors xenografted in nude mice. A549 cells were subcutaneously implanted into the 4-week-old nude mice. Three days after implantation, mice were treated with DRB18 (10mg/kg) or vehicle, IP, three times per week. After 5 weeks of treatment, mice were euthanized and tumors removed. Values shown are mean ± SEM. *P < 0.05, **P ≤ 0.01, and ***P ≤ 0.001. **a** Images of surgically removed tumors. Top row represents tumors from vehicle-treated mice and bottom represents those from DRB18-treated mice. **b** Tumor volumes (mm^3^) after 5 weeks of treatment. **c** Tumor weights (g) after 5 weeks of treatment. **d** PLS-DA analysis of metabolites in comparison between vehicle and DRB18 treated tumors. **e** Metabolite set enrichment analysis for vehicle and DRB18 treated in A549 xenograft tumors. **f** Immunofluorescence analysis of hGLUT1-4 in vehicle and DRB18 treated tumors (*n*=4). Scale bar represents 100 microns

After characterization of DRB18 as an anticancer therapeutic in vitro, we went on to test its efficacy in vivo. At the end of the DRB18 treatment, the tumors were 44% smaller by volume (Fig. 5b) and 43% smaller by weight (Fig. 5c). This study demonstrated that DRB18 was more efficacious than WZB117 which required daily injections for 10 weeks [31] and that DRB18 was administered at a frequency of 56 h between injections and a treatment duration of 5 weeks. Figure 5d and e show the PLS-DA analysis and MSEA enrichment analysis between DRB18-treated and vehicle-treated tumors. The MSEA enrichment profile shows numerous similarities in highly affected metabolic pathways (Fig. 5e) as compared with those in vitro (Fig. 3b). Qualitative assessment of DRB18 and vehicle-treated tumor sections via immunofluorescence showed DRB18 decreased expression of GLUT1-4 (Fig. 5f) and reduced proliferative capacity within the xenografted tumor, indicated by fewer Ki67-positive cells, compared with vehicle-treated samples.

No difference was found in average body weight between DRB18-treated and mock-treated tumor-bearing mice (Figure S5).

### Knockout of GLUT1 gene from A549 cells did not reduce sizes of tumors of the KO cells

**Fig. 6:**
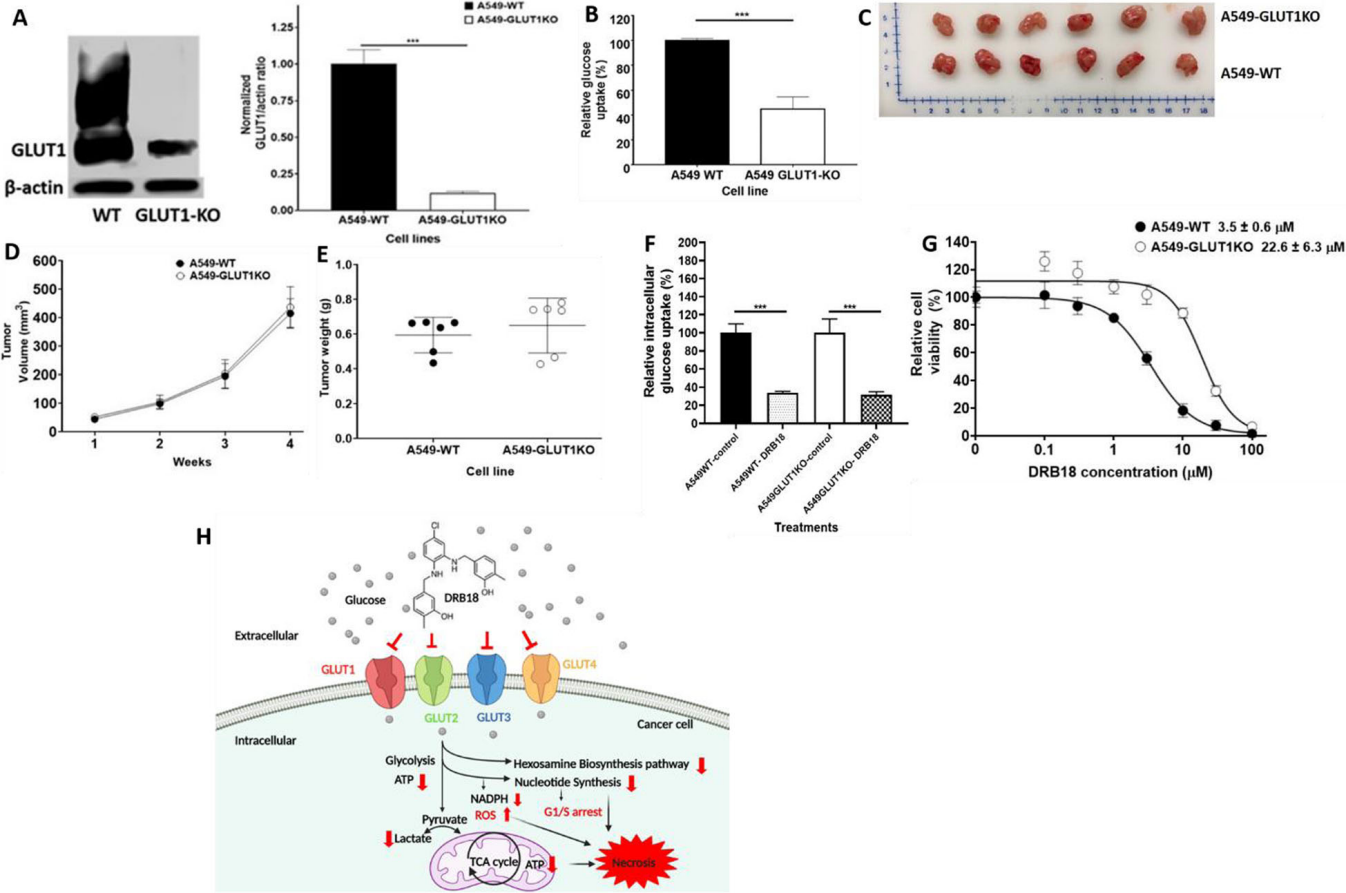
Knocking out *GLUT1* in A549 cells did not decrease xenograft tumor growth. CRISPR–Cas9-mediated *GLUT1* gene knockout was characterized by different assays. A549*GLUT1*KO tumors were generated and grown in nude mice to determine their growth rate. Values shown are mean ± SEM. *P < 0.05, **P ≤ 0.01, and ***P ≤ 0.001. **a** Western blot confirmation for GLUT1 protein knockout in A549 cells. **b** A549*GLUT1*KO cells reduced basal glucose uptake capacity compared with A549WT cells. **c** Images of tumors generated from WT and A549-*GLUT1*KO cells after 4 weeks of growth. Top row: A549*GLUT1*KO tumors, bottom row: A549-WT tumors. **d** Tumor volumes (mm^3^) after 4 weeks of growth. **e** Tumor weights (g) after 4 weeks of treatment. **f** Relative glucose intracellular glucose levels in A549 WT and A549 GLUT1KO cells treated with or without DRB18. **G**. DRB18 reduced cell viability in A549 WT and GLUT1KO cells after 72 h of treatment in a dose-dependent manner in a resazurin assay. **h** A hypothetical model for the mechanism of action of DRB18 showing it reducing glucose uptake via glucose transporters targeting glucose-based metabolism in cancer cells ultimately leading to cell death

The CRISPR-Cas9 knockout of the *GLUT1* gene led to a drastic reduction of GLUT1 protein (Fig. 6a) and a significant reduction in glucose uptake (Fig. 6b) in A549 cells. Interestingly and surprisingly, tumors generated from A549 *GLUT1*KO cells did not grow slower than the tumors generated from the original A549 cells (Fig. 6c–e), indicating that the loss of the *GLUT1* gene and its expression did not significantly alter the tumor growth rate. In addition, DRB18 treatment further reduced glucose uptake in A549*GLUT1KO* cells (Fig. 6f). The IC_50_ value for A549*GLUT1KO* cells for cell viability is about 7-fold higher than that of A549 cells (Fig. 6g). These results indicate that inhibiting a single GLUT in cancer may not work well and a pan-class I GLUT inhibition may be a logical alternative strategy for effectively inhibiting glucose transport and tumor growth.

## Discussion

Previous attempts in developing effective anti-GLUT therapeutics were predominantly focused on single GLUT targeting while most cancer cells and cancer types are multi-GLUT expressing. When one specific GLUT is inhibited, the other GLUTs and other metabolic processes could be upregulated by the cancer cells to compensate for the loss of a specific GLUT-mediated glucose transport. This could be one of the major reasons that multi-GLUT targeting may be a better alternative therapeutic solution.

The availability of four HEK293 cell lines that express only one specific GLUT per cell line ([35], Fig. 1d) made the pan-GLUT targeting study possible for DRB18. The cell line study demonstrated that DRB18 indeed inhibits glucose transport mediated by the four different class I GLUTs in the four cell lines with different IC_50_s (Fig. 1d). These different IC_50_ values in four different GLUT-expressing cell lines suggest direct binding of DRB18 to these GLUTs, since the only difference among these four cell lines is the expression of a different and unique GLUT in each cell line. In addition, the rapid glucose transport inhibition (Fig. 2b) by DRB18 also supports this conclusion. The ranking order of the GLUT glidescores (Fig. 1f) generated by the DRB18/GLUT docking study is the same as that of IC_50_ values generated by the glucose transport inhibition study in the HEK293 cell lines (Fig. 1c) with a correlation coefficient of 0.858 (Fig. 1f). The correlation further supports the notion that DRB18 directly binds to GLUT1-4. These new cell tools and new findings will significantly accelerate the development of more effective pan-class I GLUT inhibitors by synthesizing novel compounds based on computation simulation of compound-GLUT interactions.

DRB18 showed not only much improved cancer cell line inhibitions in NCI-60 screening, but it also led to tumor reduction (Fig. 5) similar to that achieved by WZB117 [31], from which DRB18 was derived. Some differences or improvements by DRB18, however, are worth mentioning. First, although the injection dosages for WZB117 and DRB18 are the same, 10 mg/kg IP, the injection frequency for WZB117 was daily (24 h per injection) while the frequency for DRB18 is three times per week (56 h per injection), a ~ 2.3-fold decrease. This change was made by taking the increased chemical stability of DRB18 over WZB117 into consideration. Second, the tumor study for WZB117 lasted for 10 weeks [31], while the DRB18 tumor study lasted for only 5 weeks. This was because the significant difference in tumor volumes between the DRB18-treated group and the mock-treated group was identified much earlier for DRB18. Further enhanced anticancer efficacy of DRB18 may be achieved by either increasing the dosage or increasing the frequency of the treatment or both. Alternatively, the improvement may be accomplished by matching inhibitors’ GLUT inhibition ratios (IC_50_s of GLUT1-4) with the GLUT-expressing ratios of specific cancer types (amount of GLUTs expressed). It is also conceivable that DRB18 and its derived pan-class I GLUT inhibitors can be used in combination with chemo or target drugs to achieve further improved anticancer efficacy with a reduced chance of generating drug resistance.

Although it is possible that some of the reduced glucose transport and glucose metabolism is due to an off-target effect, it is unlikely. First, the HEK293 cell lines’ study indicated that the IC_50_ values were different among the 4 cell lines (Fig. 1d) while the only difference among the cell lines is that they express different GLUTs [35]. This strongly suggests that the difference came from the inhibition of GLUTs by DRB18. Further study also showed that DRB18 inhibited glucose transport not only in A549 cells but also in A549 *GLUT1KO* cells (Fig. 6f), suggesting that other GLUT2-4 also participate in glucose transport in A549 cells. This result is further supported by IC_50_ comparison between A549 and A549 *GLUT1KO* cells, in which the IC_50_ of A549*GLUT1KO* cells is ~ 7-fold higher (weaker) than that of A549 cells (Fig. 6g). These results suggest that DRB18 does not impact proliferation and glucose transport in a cell line not heavily dependent on class I GLUTs, further implying that the observed effects were due to the inhibition of GLUTs, not off-targets.

The KO of the *GLUT1* gene from A549 cells did not reduce tumor volume, indicating that the tumor growth rate was not affected by the silencing of the *GLUT1* gene. This result shows that a total inhibition of a single GLUT, GLUT1 in this case, does not affect the growth of A549 tumors. The KO result provides clear experimental evidence that single GLUT targeting may not be effective and pan-GLUT targeting, as demonstrated by our DRB18 tumor study (Fig. 5a–c) is likely to be an alternative and better route for developing a new effective anticancer therapeutic strategy.

Interestingly and surprisingly, DRB18 induced a rapid but temporary increase in ECAR that persisted for about 15 min and subsided in about 30 min (Fig. 2c and d, Supplemental Figure 3). The addition of endocytosis inhibitors revealed that this increase was due to the temporary induction of macropinocytosis and clathrin-mediated endocytosis [43–45]. This is the first time macropinocytosis and clathrin-mediated endocytosis were indirectly shown to be responsible for glucose uptake in cancer cells.

The in vitro metabolomics data shows that glucose metabolism-related pathways such as glycolysis, TCA cycle, purine and pyrimidine synthesis (for biomass building blocks), and hexosamine biosynthesis (protein glycosylation) in A549 cells were significantly inhibited by the DRB18 treatment. These specific metabolic reductions correlated well with our other functional assays and provide another strong piece of evidence that DRB18 is a pan-class I GLUT inhibitor that inhibits cancer cells by reducing GLUT-mediated glucose transport and glucose metabolism. The reasons that the glycolytic metabolites did not reduce as much as anticipated are possibly related to the presence of macropinocytosis and other endocytoses (Figs. 2c and S3) as potential mechanisms by which the reduction of glycolysis was partially compensated by their internalized glucose. Other compensatory mechanisms utilizing other nutrients may also be present.

## Conclusion

Taken together, our studies described here demonstrate that DRB18 [34] is a significantly improved pan-class I GLUT inhibitor that shows more potent in vitro and in vivo anticancer efficacy compared with WZB117, the first generation of GLUT inhibitor [27–33]. The functional assays and docking study strongly suggest that DRB18 likely interacts with the outward open conformation of GLUT1-4 and then inhibits them. Metabolomics analysis combined with functional assays and immunofluorescence microscopy strongly suggest that the in vitro anticancer mechanism is primarily through inhibition of GLUT-mediated glucose uptake and its subsequent glucose metabolism-related pathways, eventually leading to, primarily, necrotic cell death. The pathways affected in vivo as revealed by the metabolomics enrichment analysis correlate with those shown in vitro, suggesting that the in vivo anticancer mechanism of DRB18 is at least in part the same as its in vitro mechanism. The *GLUT1* KO study indicates the single GLUT targeting may not work well in multi-GLUT-expressing cancer cells and a pan-GLUT approach may work much better. Figure 6f shows the hypothetical anticancer mechanism of DRB18 in cancer cells. DRB18, combined with the single GLUT-expressing cell lines, the docking study, and nude mouse tumor models, can be used as a lead compound for anticancer therapy and for designing and synthesizing even more efficacious anticancer compounds.

## Supplementary Information

**Additional file 1.** Supplementary Methods

**Additional file2: Figure S1**. DRB18 binds to GLUT2-4 in outward open conformation. **Figure S2**. DRB18 docked to hGLUT1-4 in inward open conformation. **Figure S3**. DRB18 treatment leads to induced internalization of glucose via different endocytic mechanisms. **FigureS4**. DRB18 caused G1/S phase cell cycle arrest and necrotic cell death in A549 cells. **Figure S5**. Average body weights of nude mice bearing A549 xenograft tumors from control (*n=10*) and DRB18 (*n=10*) treated groups.

## Data Availability

The data used for metabolomics analysis (both in vitro and in vivo) is available at the NIH Common Fund’s National Metabolomics Data Repository (NMDR) website, the Metabolomics Workbench, https://www.metabolomicsworkbench.org where it has been assigned Project ID PR001035 (Study ID ST001638 and ST001610). The data can be accessed directly via its Project DOI: doi: 10.21228/M8X39Q. This work is supported by NIH grant U2C-DK119886.

## Abbreviations

CHcl: Chlorpromazine hydrochloride
ECAR: Extracellular acidification rate
DMSO: Dimethyl sulfoxide
EIPA: 5-(N-Ethyl-N-isopropyl) amiloride
GLUT: Glucose transporter
GSH/GSSG: Reduced glutathione/oxidized glutathione
HBP: Hexosamine biosynthetic pathway
IP: Intraperitoneal
MSEA analysis: Metabolite set enrichment analysis
NSCLC: Non-small cell lung cancer
OCR: Oxygen consumption rate
PLS-DA: Partial least square-discriminant analysis
