# Supplementary material for: A small-molecule pan-class I glucose transporter inhibitor reduces cancer cell proliferation in vitro and tumor growth in vivo by targeting glucose-based metabolism
Source: Cancer Metab. 2021 Mar 26;9:14. doi: 10.1186/s40170-021-00248-7 (PMC8004435; doi:10.1186/s40170-021-00248-7)
Supplement: Supplementary file 1 — Additional file 1. Supplementary Methods [file 40170_2021_248_MOESM1_ESM.docx]

**Supplementary Methods**

**Protein docking studies**

The grid box settings were the same in both inward and outward open conformations for each GLUT1-4 except the centroid of the grid (Supplementary table S1). The grid box (size set for ligands <=20 Å) was centered at centroid of 3 selected amino acid residues for both inward open and outward open models with the bounding box (size 40 Å, 40 Å, 40 Å). The grids were constructed and docking performed using Glide (Schrodinger) with the OPLS3e forcefield.

| Inward open |  | Residue 1 | Residue 2 | Residue 3 |
| --- | --- | --- | --- | --- |
|  | GLUT1 | 292 Tyr | Leu 325 | 412 Trp |
|  | GLUT2 | 324 Tyr | Val 357 | 444 Trp |
|  | GLUT3 | 290 Tyr | Leu 323 | 410 Trp |
|  | GLUT4 | 308 Tyr | Val 341 | 428 Trp |
| Outward open |  |  |  |  |
|  | GLUT1 | 292 Tyr | Gln 172 | 412 Trp |
|  | GLUT2 | 324 Tyr | Gln 204 | 444 Trp |
|  | GLUT3 | 290 Tyr | Gln 170 | 410 Trp |
|  | GLUT4 | 308 Tyr | Gln 188 | 428 Trp |

Table S1. Amino acid residues used as centroid of grid box for GLUT1-4 in inward and outward open model

**LC-MS/MS metabolomics - Metabolite extraction and Sample preparation**

5 × 10^6^ A549 cells were treated with or without DRB18 (*n=3*) for 48 hours. After treatment, cells were washed twice with deionized water and polar metabolites were then extracted with cryogenically cold 80% methanol/water mixture. LC-MS grade water, methanol, and acetonitrile (Fischer Scientific, PA, USA) were used. Methanol-extracted samples were then sonicated in cycles of sonication phase and rest phase for 10 minutes (5 second sonication phase and 10 seconds halt). The samples were then centrifuged at 13,000 rpm for 10 minutes and supernatant was then collected and then lyophilization was performed.

**LC-MS/MS metabolomics - LC-MS/MS experiment and analysis**

For LC-MS/MS experiment using Agilent Q-TOF 6545 mass spectrometer, the LC gradient consisted of solvent A, H_2_O with 0.1 % Formic acid, and solvent B, 100 % acetonitrile at a 200 µL/min flow rate with an initial 2 % solvent B with a linear ramp to 95 % B at 15 min, holding at 95% B for 1 minutes, and back to 2 % B from 16 min and equilibration of 2 % B until min 32. A 5 µL volume sample was injected for each run and the top 5 ions were selected for data-dependent analysis with a 15 second exclusion window.
