# Supplementary material for: A small-molecule pan-class I glucose transporter inhibitor reduces cancer cell proliferation in vitro and tumor growth in vivo by targeting glucose-based metabolism
Source: Cancer Metab. 2021 Mar 26;9:14. doi: 10.1186/s40170-021-00248-7 (PMC8004435; doi:10.1186/s40170-021-00248-7)
Supplement: Supplementary file 2 — Additional file2: Figure S1. DRB18 binds to GLUT2-4 in outward open conformation. Figure S2. DRB18 docked to hGLUT1-4 in inward open conformation. Figure S3. DRB18 treatment leads to induced internalization of glucose via different endocytic mechanisms. FigureS4. DRB18 caused G1/S phase cell cycle arrest and necrotic cell death in A549 cells. Figure S5. Average body weights of nude mice bearing A549 xenograft tumors from control (n=10) and DRB18 (n=10) treated groups. [file 40170_2021_248_MOESM2_ESM.docx]

**Supplementary Figures**

**
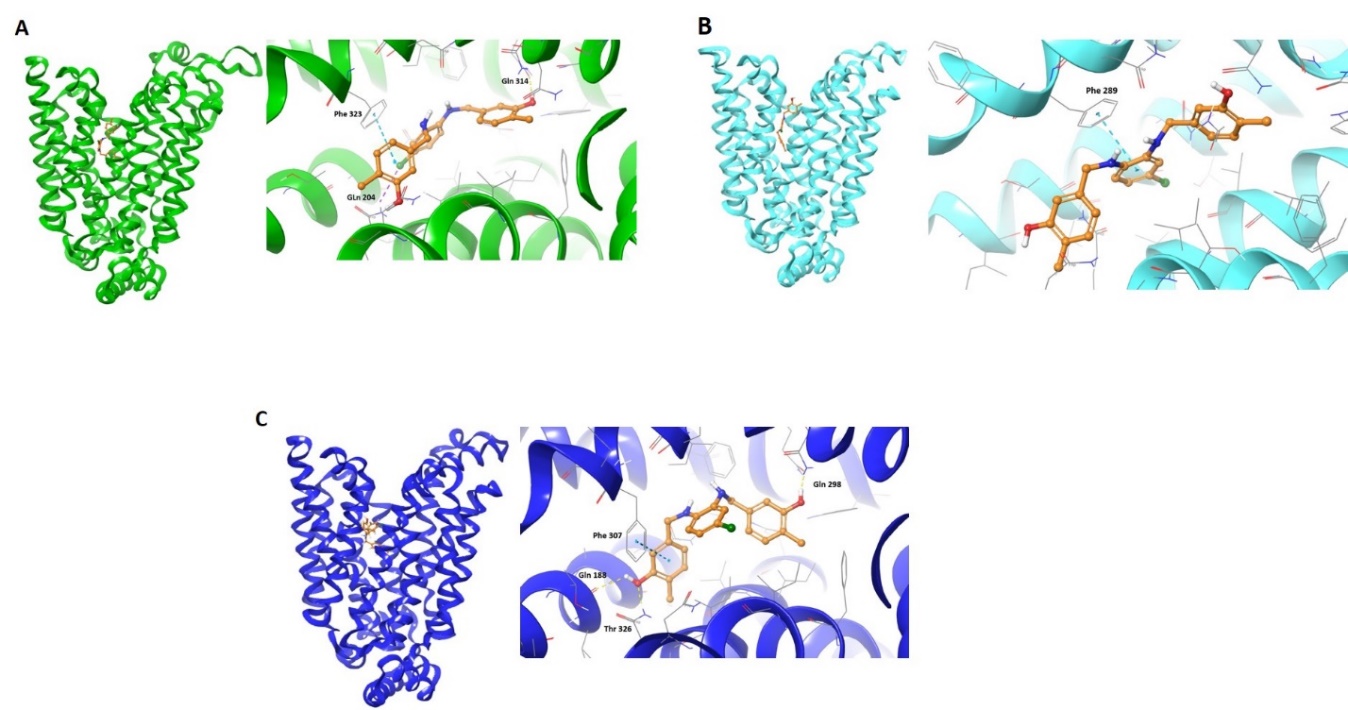
**

**Figure S1. DRB18 binds to GLUT2-4 in outward open conformation**

The protein structures for hGLUT2 and hGLUT4 were generated by homology modeling of hGLUT3 outward open model (PDB : 5C65). The proteins are shown with cytoplasmic side down. Hydrogen bonds and halogen bonds are shown in broken yellow and purple lines. π-π Interactions are shown in broken blue lines. The specific elements are shown in the respective colors with oxygen in red, nitrogen in blue and hydrogen in white. Carbons in DRB18 are shown in yellow and chlorine in dark green. Schrodinger software was used to generate the docking figures using Induced fit docking module in Glide. Glidescore were used to select the best poses. hGLUT2, color code: carbons, light green (protein); hGLUT3, color code: carbons, navy blue (protein); hGLUT4, color code: carbons, violet (protein).

1. DRB18 binds to hGLUT2 binding pocket in outward open conformation. DRB18 forms hydrogen bonds (Gln 314), π -π interactions (Phe 323) and halogen bonds (Gln 204) with different residues in hGLUT1.
2. DRB18 binds to hGLUT3 binding pocket in outward open conformation. DRB18 forms π -π interactions with (Phe 289) residue.
3. DRB18 binds to hGLUT1 binding pocket in outward open conformation. DRB18 forms hydrogen bonds with (Gln 188, Gln 298 and Thr 326) and π -π interactions (Phe 307) residues.

**
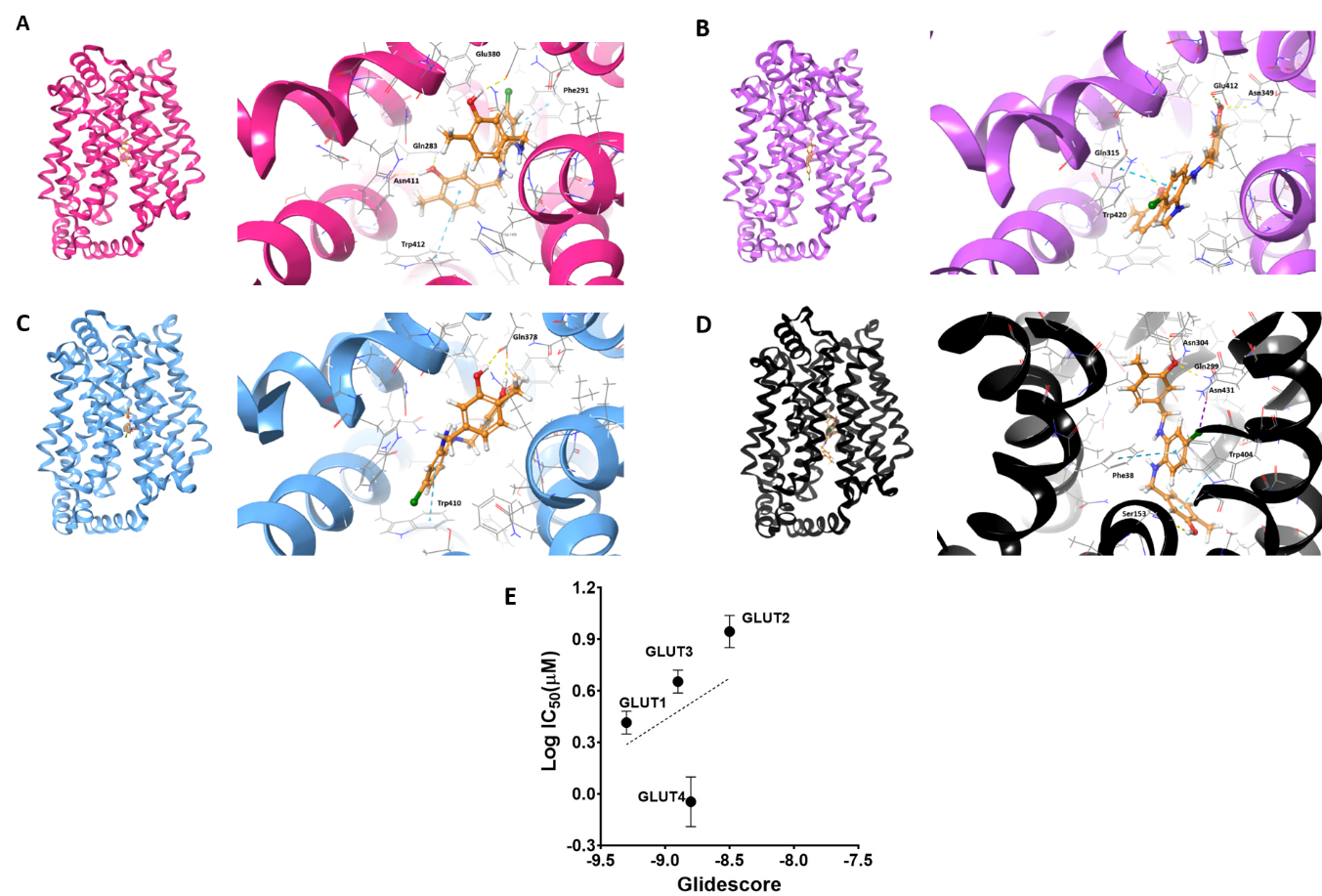
**

**Figure S2. DRB18 docked to hGLUT1-4 in inward open conformation**

The protein structures for hGLUT2-4 were generated by homology modeling of hGLUT1 inward open model (PDB : 4PYP). The proteins are shown with cytoplasmic side down. Hydrogen bonds and halogen bonds are shown in broken yellow and purple lines. π-π Interactions are shown in broken blue lines. The specific elements are shown in the respective colors with oxygen in red, nitrogen in blue and hydrogen in white. Carbons in DRB18 are shown in yellow and chlorine in dark green. Schrodinger software was used to generate the docking figures using Induced fit docking module in Glide. Glidescore were used to select the best poses. hGLUT1, color code: carbons, dark pink (protein); hGLUT2, color code: carbons, light violet (protein); hGLUT3, color code: carbons, light blue (protein); hGLUT4, color code: carbons, black (protein).

1. DRB18 docks to hGLUT1 binding pocket in inward open conformation. DRB18 forms hydrogen bonds (Gln 283, Gln 380 and Asn 411), π -π interactions (Phe 291 and Trp 412) and halogen bonds (Gln 204) with different residues in hGLUT1.
2. DRB18 docks to hGLUT2 binding pocket in inward open conformation. DRB18 forms hydrogen bonds (Gln 315, Asn 349 and Gln 412) and π -π interactions (Trp 420) with different residues in hGLUT2.
3. DRB18 docks to hGLUT3 binding pocket in inward open conformation. DRB18 forms hydrogen bonds (Gln 378) and π -π interactions (Trp 410) with different residues in hGLUT3.
4. DRB18 docks to hGLUT1 binding pocket in inward open conformation. DRB18 forms hydrogen bonds (Ser 153, Gln299 and Asn 304), π -π interactions (Phe 38 and Trp 404) and halogen bonds (Gln 431) with different residues in hGLUT4.
5. The correlation coefficient R^2^=0.1451 between experimental (glucose uptake inhibition) and virtual (Glidescore) binding suggests that DRB18 docking in the outward open conformation is better than the inward open conformation.


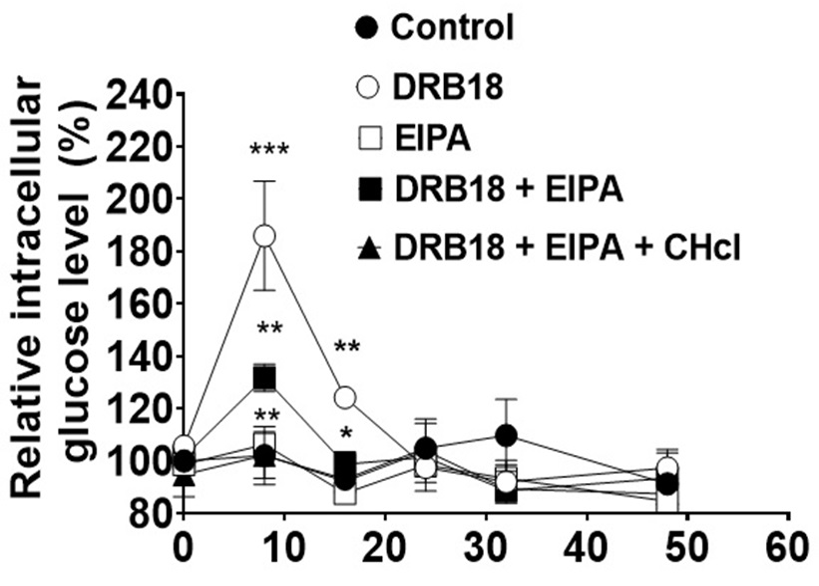


**Figure S3. DRB18 treatment leads to induced internalization of glucose via different endocytic mechanisms**

DRB18 caused a temporary increase in glucose uptake in A549 cells when experimental conditions were similar to those used in the seahorse analysis. DRB18 increased glucose internalization via in a time-dependent manner and that internalization stopped after ~24 minutes. The increase in intracellular glucose level was reduced when a macropinocytosis inhibitor EIPA was added. The increase in intracellular glucose level was completely abolished when clathrin-mediated endocytosis inhibitor Chlorpromazine hydrochloride was used along with DRB18 and EIPA.


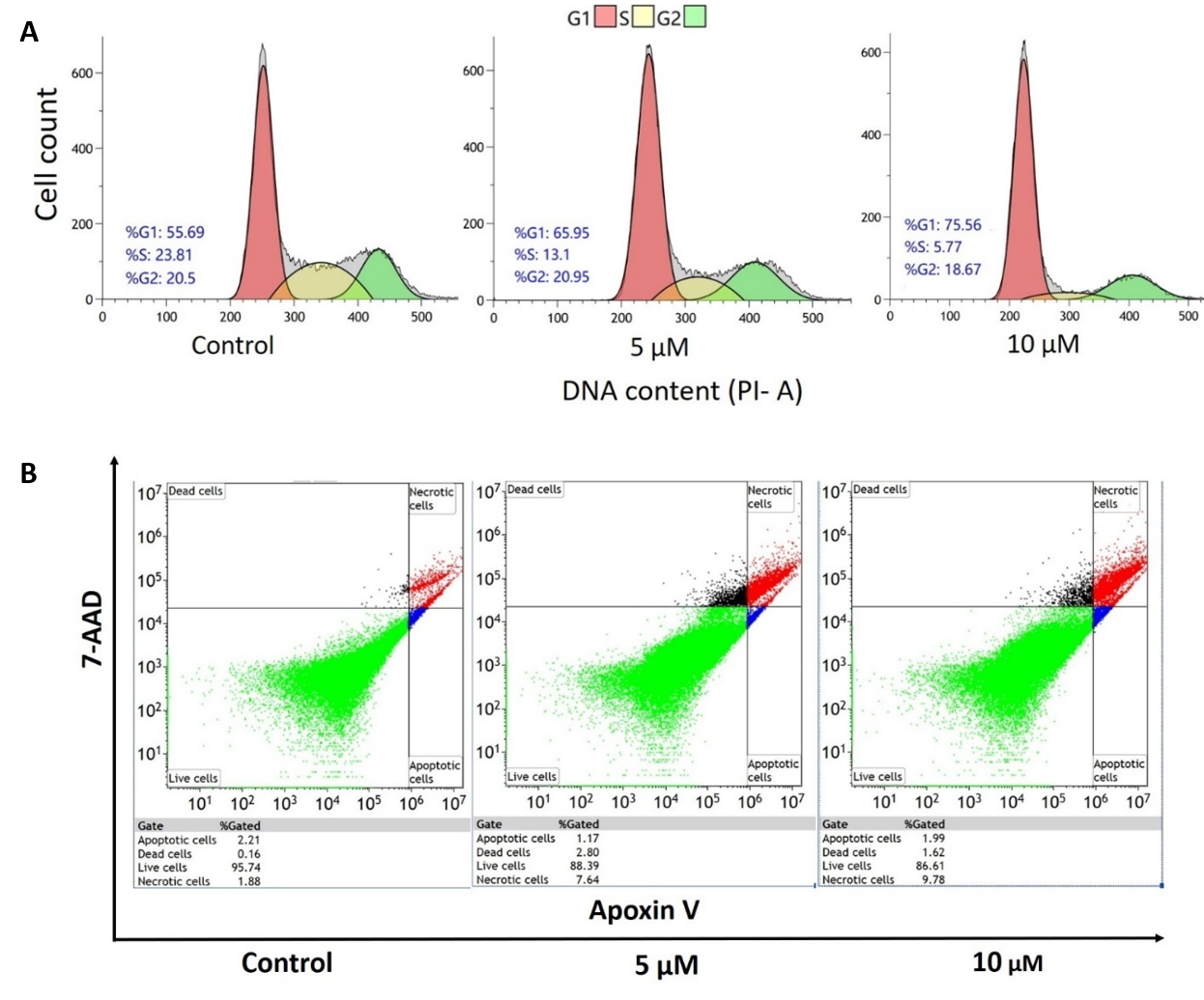


**FigureS4. DRB18 caused G1/S phase cell cycle arrest and necrotic cell death in A549 cells.**

The chronic effects of the DRB18 treatment were investigated by using cell cycle analysis and apoptosis/necrosis study in A549 cells treated or untreated with DRB18 for 72 hours.

1. DRB18 increased percentage of cells in G1 phase of cell cycle and reduces them in S-phase in a dose-dependent manner.
2. DRB18 induced necrotic cell-death in a dose-dependent manner.


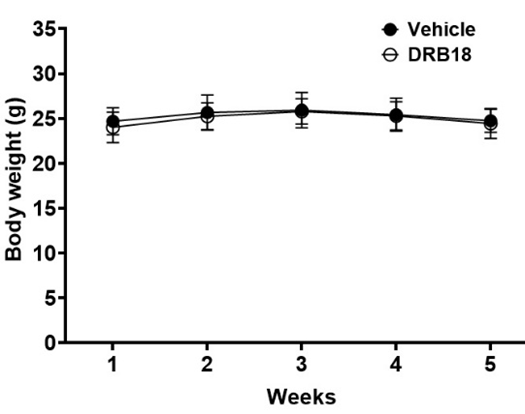


**Figure S5. Average body weights of nude mice bearing A549 xenograft tumors from control (*n=10*) and DRB18 (*n=10*) treated groups**

Average body weights of nude mice bearing A549 xenograft tumors during 5 weeks of treatment.
